# Relative versus absolute globotriaosylsphingosine changes and kidney outcomes in Fabry disease

**DOI:** 10.1016/j.ymgmr.2026.101378

**Published:** 2026-10-28

**Authors:** Toshiyuki Hirai, Shunsuke Goto, Hayaki Okamoto, Momoko Tabuchi, Mika Fujita, Hideki Fujii

**Affiliations:** Division of Nephrology, Kobe University Graduate School of Medicine, Kobe, Japan

**Keywords:** Enzyme replacement therapy, Estimated glomerular filtration rate, Fabry disease, Globotriaosylsphingosine, Urinary albumin-to-creatinine ratio

## Abstract

**Background:**

Plasma globotriaosylsphingosine (lyso-Gb3) concentrations decrease after enzyme replacement therapy (ERT) in patients with Fabry disease (FD). However, it remains unclear whether absolute or relative changes in lyso-Gb3 concentrations better reflect treatment response. We evaluated the clinical relevance of absolute and relative changes in lyso-Gb3 concentrations after ERT.

**Methods:**

This retrospective observational study included 16 patients with FD (8 males and 8 females) with elevated baseline lyso-Gb3 concentrations and paired measurements at baseline and one year after ERT. Absolute and relative changes in lyso-Gb3 concentrations were assessed, and their relationships with baseline lyso-Gb3 concentrations were examined. Kidney outcomes, including the estimated glomerular filtration rate (eGFR) slope and percent change in urinary albumin-to-creatinine ratio (UACR), were analyzed using linear mixed-effects models.

**Results:**

ERT significantly reduced plasma lyso-Gb3 concentrations in both sexes. Absolute changes in lyso-Gb3 concentrations were strongly correlated with baseline lyso-Gb3 concentrations, particularly in males, whereas relative changes showed weaker dependence on baseline concentrations. In males, larger relative reductions in lyso-Gb3 concentrations were associated with a slower decline in eGFR. Smaller relative reductions were associated with greater increases in UACR in the overall cohort, but in males this was only a nonsignificant trend.

**Conclusion:**

Relative changes in lyso-Gb3 concentrations after ERT appeared to be less influenced by baseline lyso-Gb3 concentrations than absolute changes. Associations with kidney outcomes were observed in males but were not demonstrated in females. Relative changes in lyso-Gb3 concentrations may provide additional information for assessing treatment response in FD.

## Highlights

• ERT reduced plasma lyso-Gb3 concentrations in both male and female patients.
• Relative lyso-Gb3 changes were less influenced by baseline than absolute changes.
• Larger relative lyso-Gb3 reduction was associated with slower eGFR decline in males.
• Relative lyso-Gb3 changes may better reflect kidney outcomes in males.

## 1 Introduction

Fabry disease (FD) is an X-linked lysosomal storage disorder initiated by mutations in the lysosomal enzyme α-galactosidase A (GLA)-encoding gene [1]. Deficiency of this enzyme results in globotriaosylceramide (Gb3) accumulation, a substrate of GLA, in various tissues. Globotriaosylsphingosine (lyso-Gb3), the deacylated form of Gb3, has emerged as a sensitive and disease-specific biomarker of FD and is widely used in clinical practice [2]. Progressive accumulation of Gb3 and related metabolites results in cellular dysfunction, including apoptosis, fibrosis, impaired energy metabolism, microvascular injury, oxidative stress, and tissue ischemia, ultimately resulting in multi-organ involvement, such as kidney failure, heart failure, arrhythmias, and cerebrovascular events [3]. Clinical manifestations and disease severity are influenced by the type of GLA mutation and patient sex, with males typically exhibiting more severe phenotypes than females [4]. Currently available disease-specific therapies include enzyme replacement therapies (ERT) and pharmacological chaperone therapy [5].

Among the available biomarkers, lyso-Gb3 has been shown to be more sensitive and specific than Gb3 for FD diagnosis [6]. Plasma lyso-Gb3 concentrations are often elevated even in patients with relatively mild clinical manifestations and have been reported to decrease following disease-specific treatment, including ERT [7]. In addition, previous studies have suggested that baseline lyso-Gb3 concentrations may reflect disease burden and genotype severity in FD [8]. Accordingly, lyso-Gb3 has increasingly been used as a biomarker for assessing treatment response and disease activity in both clinical practice and research settings.

However, it remains unclear whether treatment-induced reductions in lyso-Gb3 concentrations truly reflect clinically meaningful therapeutic benefits. In most previous studies, treatment response has been evaluated based on absolute changes in plasma lyso-Gb3 concentrations [9], [10]. Because baseline lyso-Gb3 concentrations vary substantially according to sex, genotype, and disease severity, the clinical significance of relative changes in lyso-Gb3 concentrations has not been fully elucidated [11]. Therefore, the present study aimed to evaluate the clinical implications of both absolute and relative changes in plasma lyso-Gb3 concentrations after ERT in patients with FD.

## 2 Methods

### 2.1 Study design and population

This retrospective observational study included consecutive patients with FD who visited our department between January 2008 and December 2023. FD was diagnosed based on reduced GLA activity and genetic testing confirming mutations in the GLA gene. A total of 31 patients, including 14 males and 17 females, were initially identified. Among these patients, six patients who did not receive ERT or who were treated with migalastat were omitted. In addition, nine patients were excluded because plasma lyso-Gb3 measurements were unavailable before and/or one year after initiation of ERT or because baseline plasma lyso-Gb3 concentrations were within the normal range. Therefore, 16 patients (8 males and 8 females) were included in the final analysis. This study was conducted in accordance with the Declaration of Helsinki and was approved by the Institutional Review Board of Kobe University Graduate School of Medicine (No. B210251). Because this was a retrospective study using existing clinical data, the requirement for written informed consent was waived by the Institutional Review Board. Information regarding the study was publicly disclosed, and patients were given the opportunity to opt out. Only de-identified data were used for analysis and reporting.

### 2.2 Data and sample collection

Clinical data, including age, sex, symptoms, past medical history, laboratory findings from blood and urine tests, echocardiography findings, electrocardiographic data, and magnetic resonance imaging results, were retrospectively collected from medical records. The estimated glomerular filtration rate (eGFR) was calculated using the Japanese equation for estimating the GFR [12], [13]. Disease severity was assessed by the weighted score (WS) applied in the FAbry STabilization indEX (FASTEX), a validated scoring system that assesses disease involvement in neurological, renal, and cardiac domains [14]. Higher scores indicate greater disease severity.

### 2.3 Measurement of α-galactosidase A activity and plasma globotriaosylsphingosine concentrations

GLA activity in leukocytes was determined by a fluorometric enzyme assay performed at a commercial laboratory (SRL Inc., Tokyo, Japan). Plasma lyso-Gb3 concentrations were measured using a validated liquid chromatography–tandem mass spectrometry (LC–MS/MS) assay at a commercial laboratory (Sekisui Medical Co., Ltd., Tokyo, Japan). The calibration range of the assay was 0.5–150 ng/mL, with a lower limit of quantification (LLOQ) of 0.5 ng/mL. The within-batch precision, expressed as the coefficient of variation (CV), ranged from 5.9% to 12.4% at the LLOQ and from 0.4% to 6.6% at the other validated concentrations. The between-batch CV was 11.9% at the LLOQ and ranged from 3.9% to 6.0% at the other validated concentrations. Samples were analyzed across multiple analytical batches.

The absolute change in plasma lyso-Gb3 concentration was calculated as the concentration at one year after ERT minus the baseline lyso-Gb3 concentration. The relative change in plasma lyso-Gb3 concentration (%) was calculated as follows: [(lyso-Gb3 concentration at one year after ERT − baseline lyso-Gb3 concentration) / baseline lyso-Gb3 concentration] × 100. Thus, negative values for both absolute and relative changes indicate reductions in lyso-Gb3 concentration after ERT, with more negative values representing larger reductions.

### 2.4 Statistical analysis

All statistical analyses were performed using IBM Statistics version 30.0 (IBM Corp., Armonk, NY, USA) and Stata/MP 14.2 (StataCorp LLC, College Station, TX, USA). Continuous variables are presented as mean ± standard deviation or median (interquartile range), as appropriate, and categorical variables as counts and percentages. Student's *t*-test and Mann–Whitney *U* test were used to compare continuous variables between males and females, and Fisher's exact test was used for categorical variables. Changes in plasma lyso-Gb3 concentrations from baseline to one year after ERT were assessed using the Wilcoxon signed-rank test. Associations between baseline lyso-Gb3 concentrations and absolute or relative changes in lyso-Gb3 concentrations were estimated using Spearman's rank correlation analysis. Patients were stratified into two groups according to the sex-specific median values of absolute or relative changes in lyso-Gb3 concentrations. For analyses of the overall cohort, including both between-group comparisons and linear mixed-effects models, patients were first classified according to the median value within their respective sex and were then combined across sexes. Differences between groups were assessed using the Mann–Whitney *U* test. Linear mixed-effects models were used to evaluate the associations between lyso-Gb3 changes and kidney outcomes. For eGFR slope analysis, eGFR was modeled as the dependent variable with fixed effects for group, time since ERT initiation, and their interaction, and the eGFR slope was estimated from the coefficient of the time variable. For urinary albumin-to-creatinine ratio (UACR) analysis, the percentage change in UACR from baseline was used as the dependent variable, with group included as a fixed effect. All models were adjusted for age, sex, baseline brain natriuretic peptide, type of ERT regimen, use of renin–angiotensin system inhibitors, and sodium–glucose cotransporter 2 inhibitors. A two-sided *P* value less than 0.05 was considered statistically significant.

## 3 Results

### 3.1 Baseline characteristics

Five GLA variants were identified among the study population: c.281G>A (*n* = 7), c.928C>T (*n* = 4), c.658C>T (*n* = 2), c.880T>G (*n* = 2), and c.695T>C (*n* = 1). The c.695T>C variant has been previously reported to be associated with a late-onset phenotype of FD, whereas the remaining variants are associated with the classical phenotype. The baseline clinical characteristics and laboratory findings of the participants are summarized in Table 1. Compared with males, females were significantly older, had higher WS, and exhibited lower eGFR. In contrast, males showed significantly lower GLA activity and markedly higher plasma lyso-Gb3 concentrations than females. Among the eight male patients, five received agalsidase α and three received agalsidase β; among the eight female patients, three received agalsidase α and five received agalsidase β.

**Table 1:** Baseline characteristics.

	Total (*n* = 16)	Males (*n* = 8)	Females (*n* = 8)	*P* value
Age (yrs)	38.3 ± 19.5	23.3 ± 12.3	53.4 ± 12.1	<0.001
Stroke (*n*, %)	1 (6.3)	0 (0)	1 (12.5)	1.000
Acroparesthesia (*n*, %)	7 (43.8)	5 (62.5)	2 (25.0)	0.315
Hypohidrosis (*n*, %)	7 (43.8)	5 (62.5)	2 (25.0)	0.315
Angiokeratoma (*n*, %)	14 (87.5)	8 (100)	6 (75.0)	0.467
Corneal opacities (*n*, %)	15 (93.8)	7 (87.5)	8 (100)	1.000
Weighted score (%)	59.4 ± 26.5	43.2 ± 25.3	75.5 ± 16.5	0.009
eGFR (mL/min/1.73 m^2^)	93.7 ± 35.7	117.9 ± 35.2	69.5 ± 12.7	0.003
UACR (mg/gCr)	49.9 (12.6–188.7)	18.3 (12.6–63.7)	87.5 (26.2–865.9)	0.234
BNP (pg/mL)	19.54 (8.33–168.57)	12.15 (7.39–25.41)	114.15 (19.19–374.10)	0.065
ERT, Agalsidase α/β (*n*)	8/8	5/3	3/5	0.619
GLA (nmol/mg protein/h)	3.60 (0.10–34.73)	0.10 (0.03–0.40)	34.25 (26.05–43.38)	<0.001
Baseline lyso-Gb3 (ng/mL)	38.65 (10.65–177.50)	176.00 (82.85–205.75)	10.70 (6.87–31.73)	0.001

eGFR, estimated glomerular filtration rate; UACR, urinary albumin-to-creatinine ratio; BNP, brain natriuretic peptide; ERT, enzyme replacement therapy; GLA, α-Galactosidase A; lyso-Gb3, globotriaosylsphingosine.

### 3.2 Changes in globotriaosylsphingosine concentrations before and one year after enzyme replacement therapy

Plasma lyso-Gb3 concentrations decreased significantly one year after initiation of ERT in both males and females (Fig. 1). In males, plasma lyso-Gb3 concentrations were substantially reduced compared with baseline concentrations. In females, despite lower and more heterogeneous baseline lyso-Gb3 concentrations, a significant reduction was also observed one year after ERT. When expressed relative to baseline concentrations, plasma lyso-Gb3 concentrations showed a consistent pattern of decline across both sexes. Notably, relative changes appeared to provide a more uniform comparison among patients with different baseline lyso-Gb3 concentrations, particularly in females, in whom absolute changes demonstrated greater variability.

**Fig. 1:**
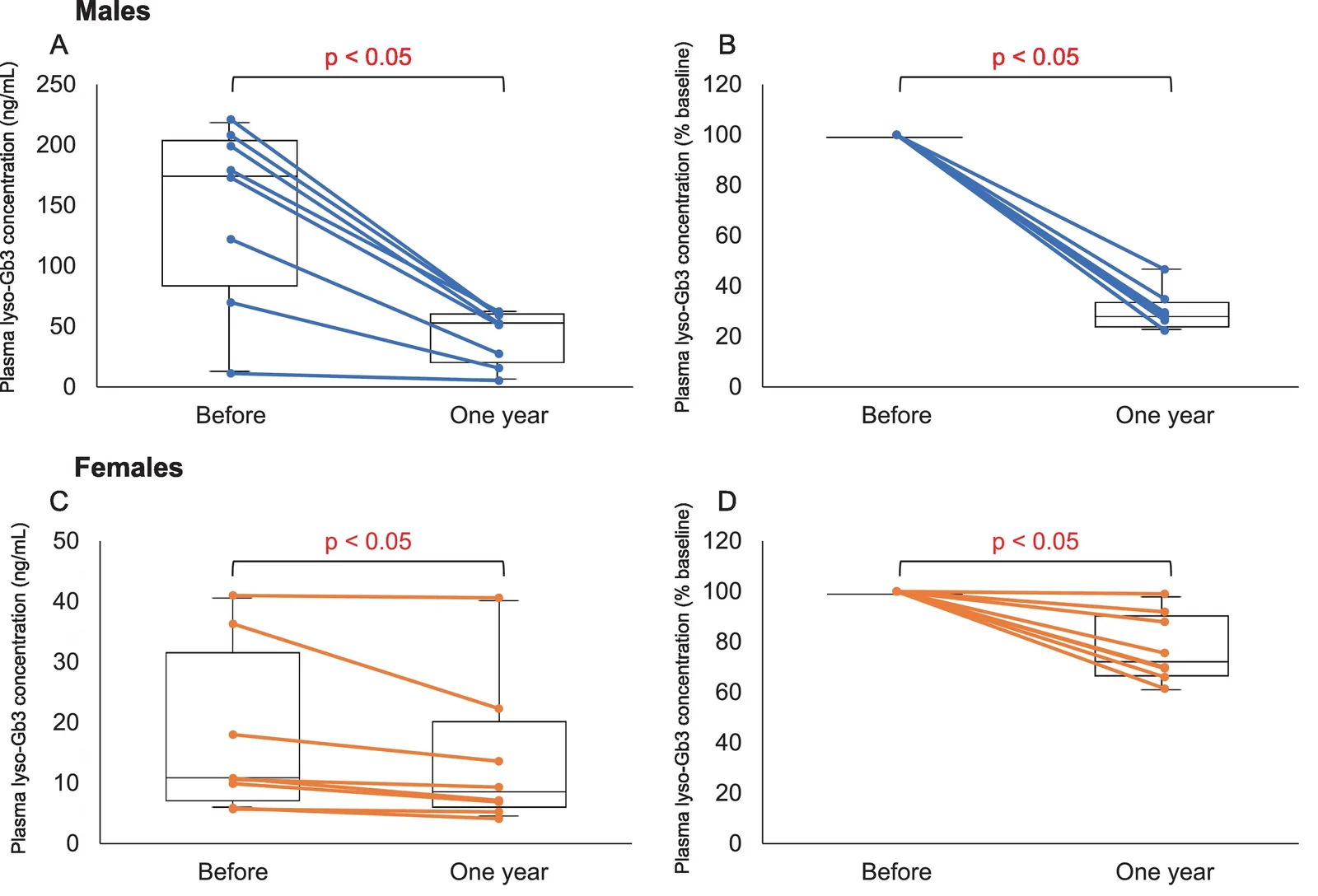
Changes in plasma globotriaosylsphingosine (lyso-Gb3) concentrations from baseline to one year after enzyme replacement therapy (ERT), stratified by sex. (A, B) Males and (C, D) females.

### 3.3 Association between baseline globotriaosylsphingosine concentrations and changes in globotriaosylsphingosine concentrations after enzyme replacement therapy

In the overall cohort, both absolute and relative changes in plasma lyso-Gb3 concentrations were significantly correlated with baseline lyso-Gb3 concentrations, with a stronger correlation observed for absolute changes than for relative changes (Fig. 2). In males, absolute changes in lyso-Gb3 concentrations demonstrated a strong and significant correlation with baseline lyso-Gb3 concentrations, whereas relative changes showed no significant correlation. In females, neither absolute nor relative changes in lyso-Gb3 concentrations were significantly correlated with baseline lyso-Gb3 concentrations. When patients were stratified into two groups according to median baseline lyso-Gb3 concentrations, comparisons of changes in lyso-Gb3 concentrations between the low and high-baseline lyso-Gb3 groups demonstrated similar trends (Fig. 3).

**Fig. 2:**
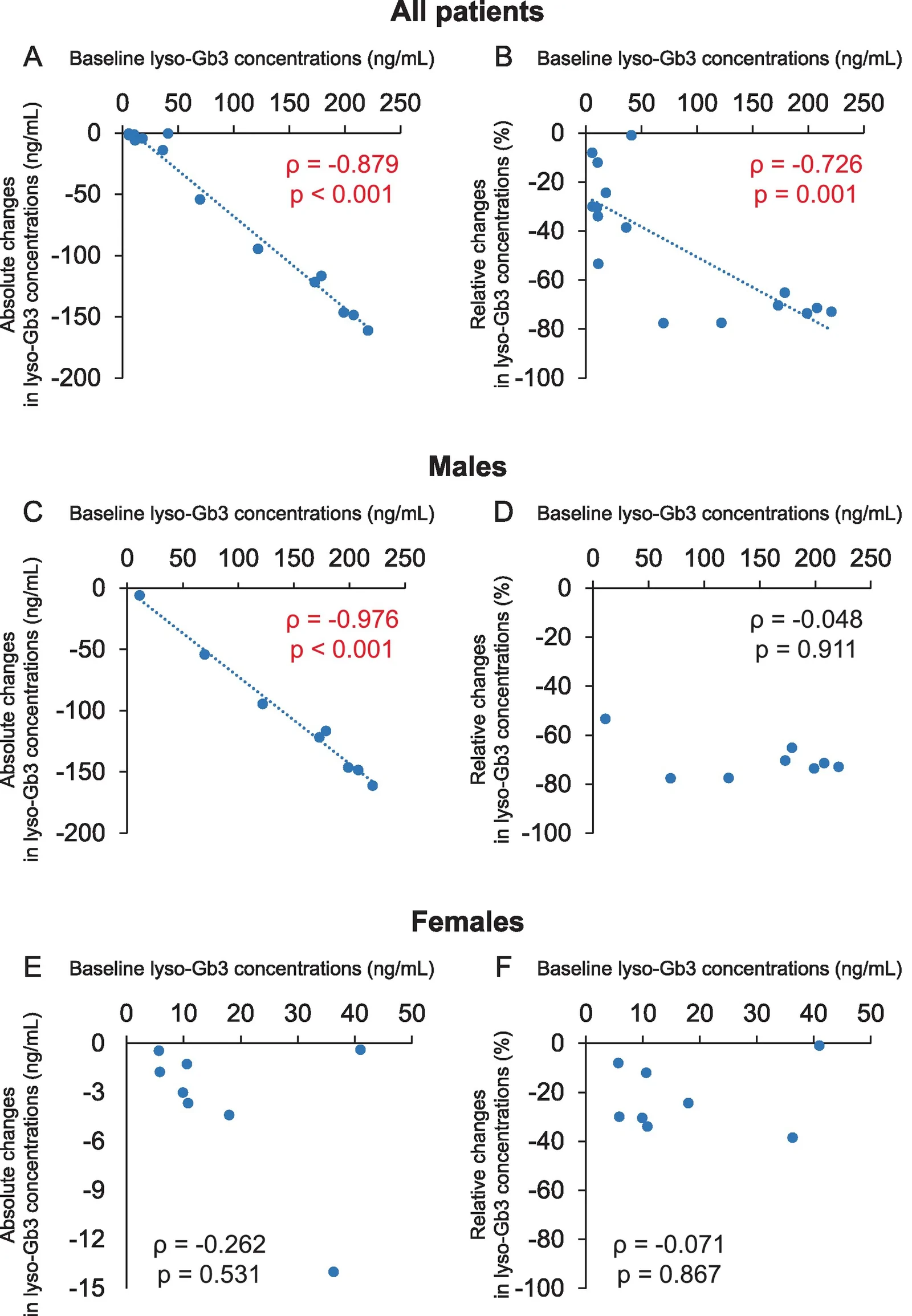
Correlations between baseline globotriaosylsphingosine (lyso-Gb3) concentrations and absolute or relative changes in lyso-Gb3 concentrations from baseline to one year after enzyme replacement therapy (ERT). (A, B) All patients, (C, D) males, and (E, F) females.

**Fig. 3:**
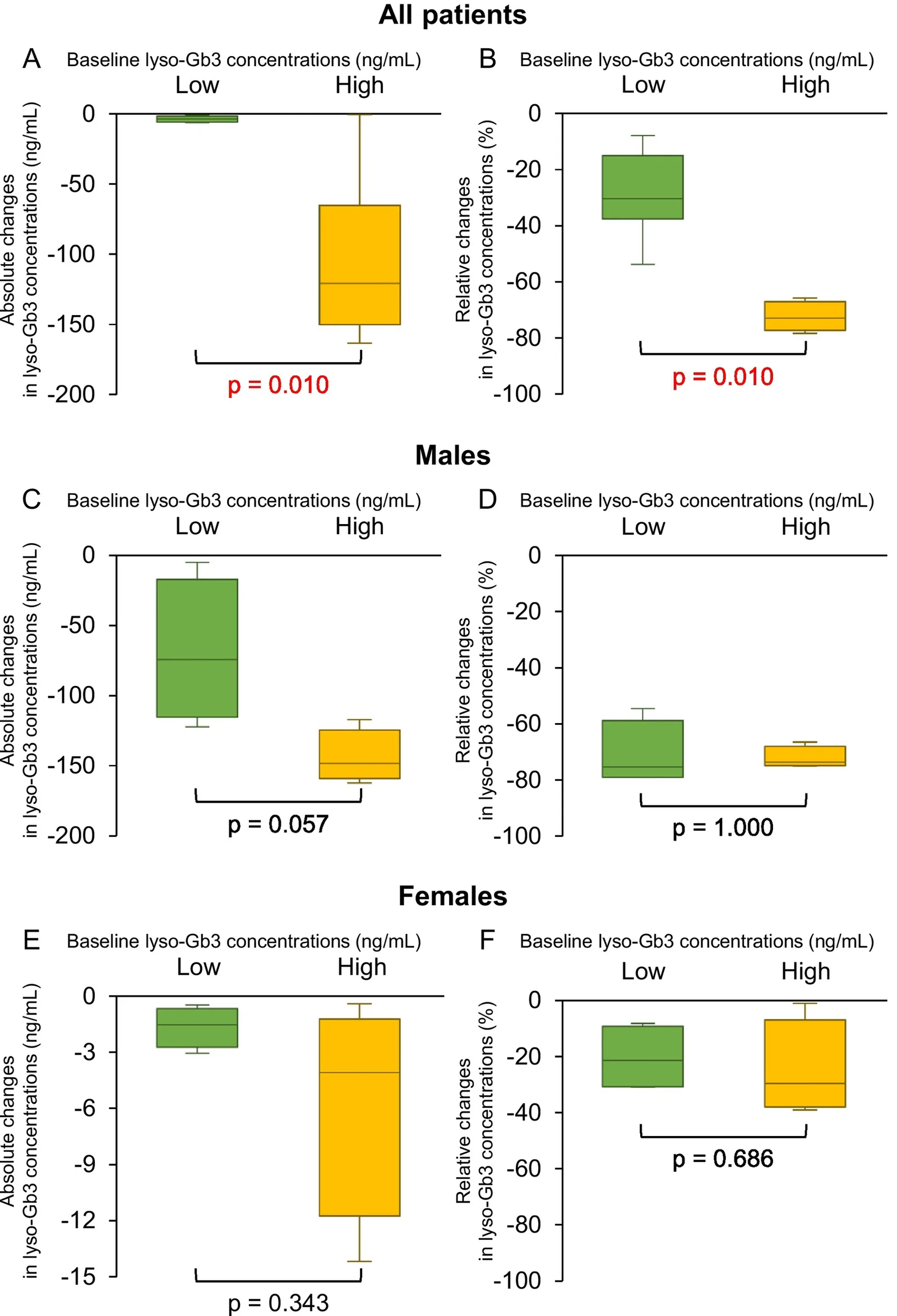
Comparisons of absolute or relative changes in globotriaosylsphingosine (lyso-Gb3) concentrations between patients with lower versus higher baseline lyso-Gb3 concentrations. (A, B) All patients, (C, D) males, and (E, F) females.

### 3.4 Association of estimated glomerular filtration rate slopes and urinary albumin-to-creatinine ratio with changes in globotriaosylsphingosine concentrations after enzyme replacement therapy

Fig. 4 shows adjusted eGFR slopes in groups stratified according to sex-specific median changes in lyso-Gb3 concentrations after ERT. In the overall cohort, no significant differences in eGFR slopes were observed between groups stratified by either absolute or relative reductions in lyso-Gb3 concentrations. In males, patients with larger absolute reductions in lyso-Gb3 concentrations exhibited a steeper decline in eGFR, whereas those with larger relative reductions showed a slower decline in eGFR. In females, eGFR slopes did not differ according to either absolute or relative reductions in lyso-Gb3 concentrations.

**Fig. 4:**
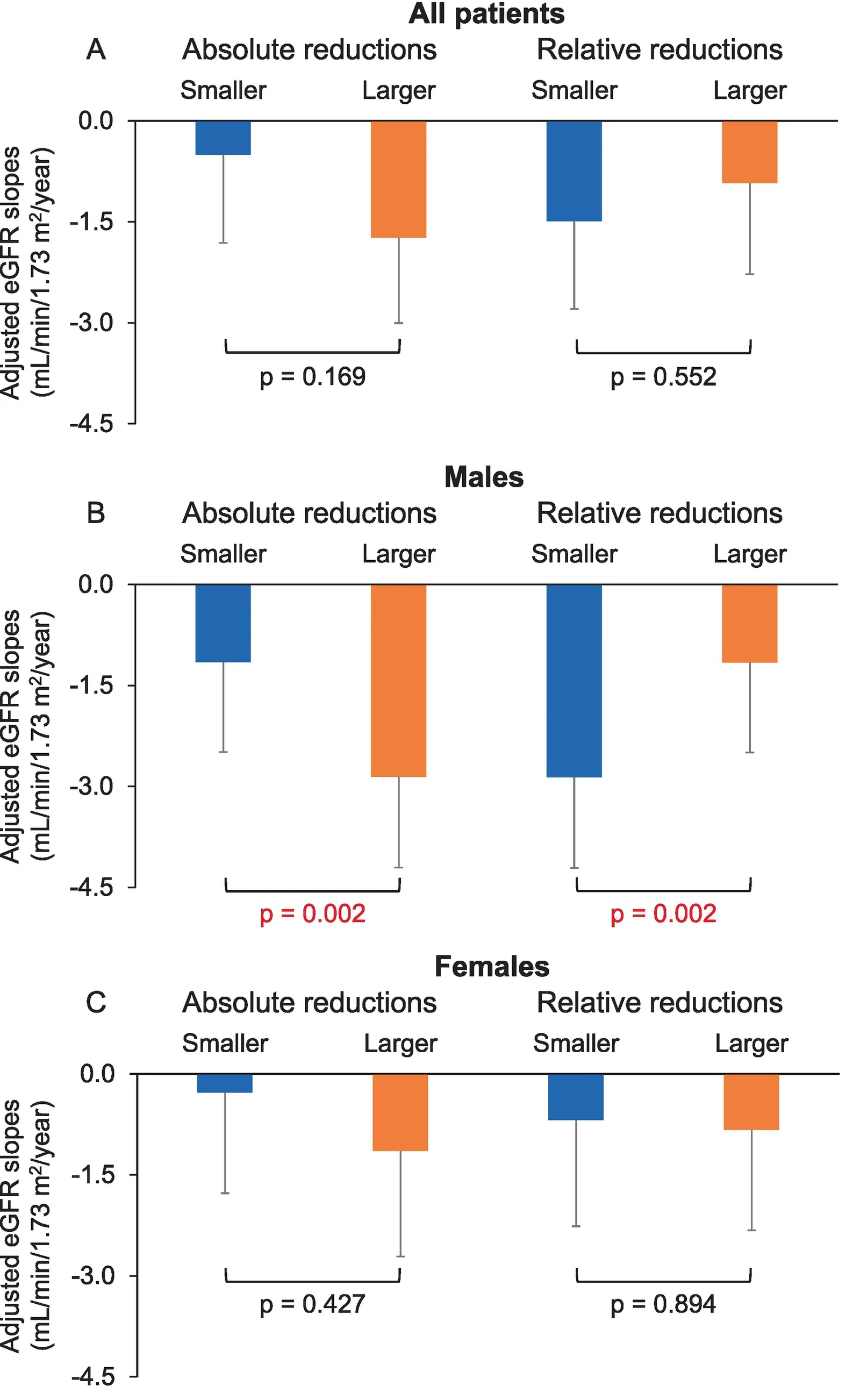
Adjusted estimated glomerular filtration rate (eGFR) slopes according to absolute or relative reductions in globotriaosylsphingosine (lyso-Gb3) concentrations from baseline to one year after enzyme replacement therapy (ERT), estimated using linear mixed-effects models. (A) All patients, (B) males, and (C) females.

We also evaluated the association between the percentage change in UACR from baseline and groups stratified by absolute or relative reductions in lyso-Gb3 concentrations (Fig. 5). The group with larger absolute reductions in lyso-Gb3 concentrations showed a greater percentage increase in UACR than the group with smaller absolute reductions. In contrast, the group with smaller relative reductions in lyso-Gb3 concentrations exhibited a greater percentage increase in UACR than the group with larger relative reductions. In males, larger absolute reductions in lyso-Gb3 concentrations were significantly associated with a greater increase in UACR, whereas smaller relative reductions showed a nonsignificant trend toward a greater increase in UACR (*P* = 0.093). No significant associations between UACR and reductions in lyso-Gb3 concentrations were observed in females.

**Fig. 5:**
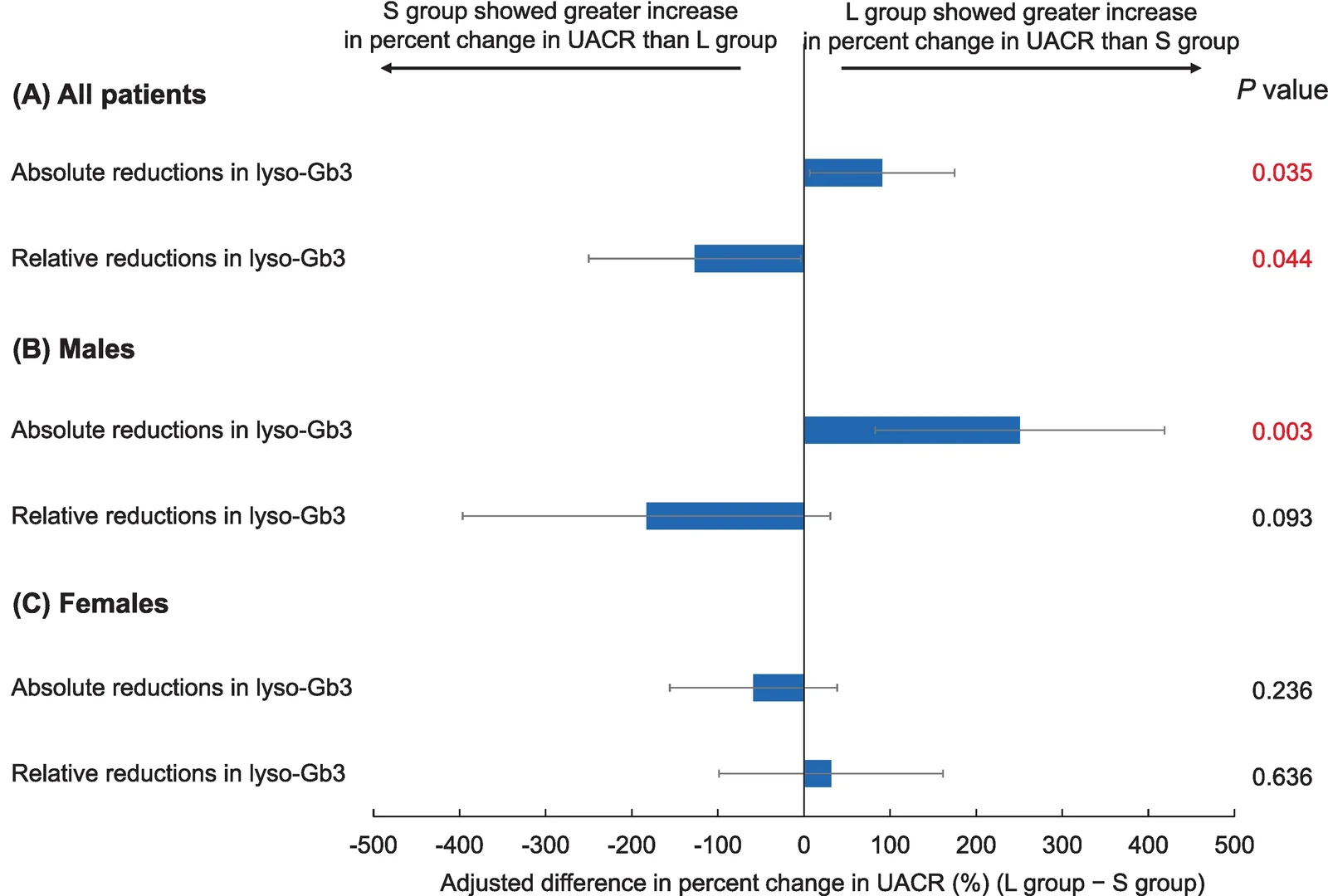
Adjusted percent change in urinary albumin-to-creatinine ratio (UACR) according to absolute or relative reductions in globotriaosylsphingosine (lyso-Gb3) concentrations from baseline to one year after enzyme replacement therapy (ERT), estimated using linear mixed-effects models. (A) All patients, (B) males, and (C) females. L group, group with larger reductions in lyso-Gb3 concentrations; S group, group with smaller reductions in lyso-Gb3 concentrations.

## 4 Discussion

In this study, we identified several key findings. First, ERT significantly reduced plasma lyso-Gb3 concentrations in both male and female patients, and relative changes provided a more uniform representation of treatment response across patients with heterogeneous baseline concentrations. Second, although both absolute and relative changes in lyso-Gb3 concentrations were significantly correlated with baseline lyso-Gb3 concentrations in the overall cohort, sex-stratified analyses demonstrated that significant correlations were detected only for absolute changes in male patients, whereas relative changes were not significantly associated with baseline lyso-Gb3 concentrations in either sex. Third, among male patients, larger absolute reductions in lyso-Gb3 concentrations were significantly associated with a steeper decline in eGFR, whereas larger relative reductions were significantly associated with a slower decline in eGFR. Fourth, in the overall cohort, larger absolute reductions in lyso-Gb3 concentrations were associated with greater percent increases in UACR, whereas smaller relative reductions were associated with greater percent increases in UACR.

Lyso-Gb3 concentrations are known to be elevated in patients with FD and to decrease after ERT [9], [10], [15]. However, both baseline lyso-Gb3 concentrations and the magnitude of absolute reductions after ERT differ substantially between males and females. Plasma lyso-Gb3 concentrations are substantially lower in females than in males; accordingly, absolute reductions in lyso-Gb3 concentrations after ERT also tend to be smaller in females [16]. Consistent with these observations, our study demonstrated statistically significant absolute reductions in lyso-Gb3 concentrations after ERT in both sexes. However, as illustrated in Fig. 1, considerable interindividual variability in baseline lyso-Gb3 concentrations was observed, particularly among females, which may complicate interpretation of treatment response when assessed solely by absolute changes. Importantly, previous studies have shown that lyso-Gb3 concentrations in females with FD remain elevated relative to healthy controls [17] and correlate with disease severity indices such as the Mainz Severity Score Index (MSSI) [18], supporting the clinical relevance of this biomarker. In contrast, relative reductions normalized to baseline concentrations were more consistent across patients, enabling a more direct comparison of treatment response regardless of baseline heterogeneity. These findings suggest that evaluating ERT efficacy based solely on absolute changes in lyso-Gb3 concentrations may be limited, particularly in patients with low baseline concentrations, such as females. Overall, our results suggest that relative changes in lyso-Gb3 concentrations may provide complementary information regarding ERT-induced biochemical responses in these populations.

In our study, absolute changes in lyso-Gb3 concentrations exhibited a strong negative correlation with baseline lyso-Gb3 concentrations in the overall cohort and in male patients. This finding suggests that male patients with higher baseline lyso-Gb3 concentrations tend to experience larger absolute reductions in lyso-Gb3 concentrations after ERT. Such individuals may represent a higher-risk population, as elevated baseline lyso-Gb3 concentrations have been associated with an increased risk of adverse long-term outcomes [19]. Although elevated lyso-Gb3 concentrations are recognized as a prognostic risk factor in patients with FD, reductions in lyso-Gb3 concentrations have not consistently correlated with improvements in clinical outcomes [20]. This discrepancy may partly reflect the strong dependence of absolute changes in lyso-Gb3 concentrations on baseline values. In contrast, the association between baseline lyso-Gb3 concentrations and relative changes was weaker. Importantly, although relative changes were significantly associated with baseline lyso-Gb3 concentrations in the overall cohort, this association was not statistically significant in either males or females when analyzed separately. Given the substantial differences in baseline lyso-Gb3 concentrations between males and females, the association observed in the overall cohort may partly reflect between-sex differences rather than a true within-sex relationship. Accordingly, the results of the pooled analyses should be interpreted with caution. Taken together, these findings suggest that relative changes may be less dependent on baseline lyso-Gb3 concentrations than absolute changes and may provide a complementary metric for assessing biochemical response to ERT.

In our study, larger absolute reductions in lyso-Gb3 concentrations were not associated with improved kidney outcomes, whereas larger relative reductions tended to be associated with more favorable kidney outcomes, particularly in males. Importantly, these associations were not demonstrated in females. To our knowledge, no previous studies have demonstrated a clear association between reductions in lyso-Gb3 concentrations and kidney outcomes. One clinical study of migalastat therapy reported no association between changes in lyso-Gb3 concentrations and changes in eGFR; however, lyso-Gb3 changes in that study were evaluated only as absolute changes [21]. Another study reported that neither absolute nor relative changes in lyso-Gb3 concentrations were associated with clinical events, including kidney events [22]. To date, few studies have specifically investigated the relationship between changes in lyso-Gb3 concentrations and longitudinal kidney function in patients with FD. Therefore, the observed association between relative reductions in lyso-Gb3 concentrations and kidney outcomes in males should be considered hypothesis-generating and requires validation in larger, prospective cohorts, including adequate numbers of both male and female patients.

Several limitations of this study should be acknowledged. First, the sample size was relatively small, particularly in the sex-stratified analyses, which included only eight patients in each sex group, and multiple exploratory comparisons were performed without formal correction for multiple comparisons. Therefore, limited statistical power as well as an increased possibility of Type I error should be considered. This is especially important because previous larger and longer-term studies have not consistently demonstrated an association between treatment-induced reductions in lyso-Gb3 concentration and clinical outcomes. Accordingly, the associations observed in the present study should be regarded as hypothesis-generating and require confirmation in larger, independent cohorts with longer follow-up. Nevertheless, given the rarity of FD, recruitment of a large cohort remains challenging. Second, this was a single-center retrospective study, which may have introduced selection bias and limits the generalizability of the findings to broader FD populations. Third, anti-ERT antibody status was not systematically available in this cohort. Because anti-drug antibodies, particularly in males with classical FD, can attenuate biochemical responses to ERT and may influence clinical outcomes [10], the inability to account for antibody status represents an important potential source of residual confounding. Fourth, lyso-Gb3 concentrations were assessed only at baseline and one year after initiation of ERT, and longer-term trajectories could not be assessed. Fifth, the type of ERT was not randomly assigned and differed among patients. Although ERT regimen was included as a covariate in the adjusted analyses, residual confounding related to treatment selection or differential biochemical responses to agalsidase α and β cannot be excluded. Sixth, as most patients in this cohort had the classical phenotype, the generalizability of the findings to patients with the late-onset phenotype remains uncertain. Despite these limitations, a major strength of this study is the concurrent assessment of both absolute and relative changes in lyso-Gb3 concentrations, an approach that has been applied in only a limited number of previous studies.

In conclusion, relative changes in lyso-Gb3 concentrations after ERT appeared to be less influenced by baseline lyso-Gb3 concentrations than absolute changes. Associations with kidney outcomes were observed in males but were not demonstrated in females. Relative changes in lyso-Gb3 concentrations may provide complementary information regarding biochemical response to ERT; however, these exploratory findings require confirmation in larger prospective studies including both male and female patients.

## Studies in human

This study was performed in compliance with relevant laws, regulatory frameworks and guidelines where the research took place.

This study was approved by the Institutional Review Board of Kobe University Graduate School of Medicine (Approval No. B210251).

## CRediT authorship contribution statement

**Toshiyuki Hirai:** Writing – original draft. **Shunsuke Goto:** Formal analysis. **Hayaki Okamoto:** Data curation. **Momoko Tabuchi:** Data curation. **Mika Fujita:** Data curation. **Hideki Fujii:** Writing – review & editing.

## Informed consent and patient details

The authors have NOT obtained informed consent from participants or their legal representatives. This was a retrospective observational study using existing clinical data. The Institutional Review Board waived the requirement for individual written informed consent in accordance with the approved study protocol. Information about the study was made publicly available, and patients were given the opportunity to opt out of participation. Only de-identified data were used for the analysis.

## Funding

This research did not receive any specific grant from funding agencies in the public, commercial, or not-for-profit sectors.

## Declaration of competing interest

The authors declare the following financial interests/personal relationships which may be considered as potential competing interests: Hideki Fujii has received lecture fees from Sumitomo Pharma, Takeda Pharmaceutical Company, Amicus Therapeutics, and Sanofi, and research support from JCR Pharma. Hayaki Okamoto has received lecture fees from Sumitomo Pharma, Takeda Pharmaceutical Company, Amicus Therapeutics, and Sanofi. The remaining autohrs declare that they have no known competing financial interests or personal relationships that could have appeared to influence the work reported in this paper.

## Data Availability

Data will be made available on request.
